# Preclinical TSPO Ligand PET to Visualize Human Glioma Xenotransplants: A Preliminary Study

**DOI:** 10.1371/journal.pone.0141659

**Published:** 2015-10-30

**Authors:** Jason R. Buck, Eliot T. McKinley, Allie Fu, Ty W. Abel, Reid C. Thompson, Lola Chambless, Jennifer M. Watchmaker, James P. Harty, Michael K. Cooper, H. Charles Manning

**Affiliations:** 1 Vanderbilt University Institute of Imaging Science (VUIIS), Vanderbilt University Medical Center, Nashville, TN, United States of America; 2 Department of Medicine, Vanderbilt University Medical Center, Nashville, TN, United States of America; 3 Department of Pathology, Vanderbilt University Medical Center, Nashville, TN, United States of America; 4 Vanderbilt-Ingram Cancer Center (VICC), Vanderbilt University Medical Center, Nashville, TN, United States of America; 5 Department of Neurological Surgery, Vanderbilt University Medical Center, Nashville, TN, United States of America; 6 Program in Chemical and Physical Biology, Vanderbilt University Medical Center, Nashville, TN, United States of America; 7 Department of Radiology and Radiological Sciences, Vanderbilt University Medical Center, Nashville, TN, United States of America; 8 Neurology Service, Veterans Affairs Tennessee Valley Healthcare System, Nashville, TN, United States of America; 9 Department of Neurology, Vanderbilt University Medical Center, Nashville, TN, United States of America; University of Manchester, UNITED KINGDOM

## Abstract

Current positron emission tomography (PET) imaging biomarkers for detection of infiltrating gliomas are limited. Translocator protein (TSPO) is a novel and promising biomarker for glioma PET imaging. To validate TSPO as a potential target for molecular imaging of glioma, TSPO expression was assayed in a tumor microarray containing 37 high-grade (III, IV) gliomas. TSPO staining was detected in all tumor specimens. Subsequently, PET imaging was performed with an aryloxyanilide-based TSPO ligand, [^18^F]PBR06, in primary orthotopic xenograft models of WHO grade III and IV gliomas. Selective uptake of [^18^F]PBR06 in engrafted tumor was measured. Furthermore, PET imaging with [^18^F]PBR06 demonstrated infiltrative glioma growth that was undetectable by traditional magnetic resonance imaging (MRI). Preliminary PET with [^18^F]PBR06 demonstrated a preferential tumor-to-normal background ratio in comparison to 2-deoxy-2-[^18^F]fluoro-D-glucose ([^18^F]FDG). These results suggest that TSPO PET imaging with such high-affinity radiotracers may represent a novel strategy to characterize distinct molecular features of glioma growth, as well as better define the extent of glioma infiltration for therapeutic purposes.

## Introduction

Malignant gliomas are characterized by invasive growth and recalcitrance to current therapies. In contrast to contemporary precision cancer medicine, magnetic resonance imaging (MRI) remains the standard radiographic modality used for brain tumor imaging and is primarily used to determine the extent of tumor involvement pre- and post-treatment. MRI approximates the tumor core by examining the amount of solid tumor enhancement on T_1_-weighted post-contrast images, but poorly evaluates the amount of microscopic tumor infiltration, which is sometimes estimated by increased signal on T_2_-weighted and T_2_-FLAIR sequences. T_2_-weighted and T_2_-FLAIR sequences demonstrate peri-tumoral hyperintense signals that are non-specific and do not distinguish microscopic neoplastic infiltration from edema, myelin loss, or gliosis. These imaging constraints impact the accuracy of diagnostic biopsies and optimal therapeutic utilizations of surgery, radiotherapy, and chemotherapy. For example, a gross total resection is currently defined by surgical removal of all enhancing tumor, despite the understanding that invasive tumor remains. Additionally, accurate interpretation of MRI surveillance studies are complicated by the effects of radiation, surgery, and bevacizumab, all treatments that can cause abnormal enhancement and hyperintense T_2_-weighted and T_2_-FLAIR signal changes that are indistinguishable from residual tumor on imaging [1].

While MRI reveals anatomical details, it lacks molecular information specific to glioma growth and metabolism. As an alternative, there is positron emission tomography (PET), a functional imaging technique that enables highly sensitive measurement of molecular processes using radiotracers labeled with positron-emitting isotopes (carbon-11/^11^C; fluorine-18/^18^F). The increased sensitivity of PET, coupled with the ability to produce biologically active tracers bearing PET imaging isotopes, could potentially enable the detection of tumor and small clusters of cells at infiltrative/invasive margins [2].

Currently, the FDA-approved glucose analog 2-deoxy-2-[^18^F]fluoro-D-glucose ([^18^F]FDG) is the most widely applied radiotracer for brain tumor assessment [3, 4]. While [^18^F]FDG accumulates in tissues exhibiting elevated glucose metabolism [5], high glucose uptake in normal brain can result in poor tumor-to-background ratios that can confound adequate glioma detection. Radiolabeled amino acids such as [^11^C]MET (L-methyl-[^11^C]-methionine) have been developed as alternatives, and while these agents target tissues exhibiting enhanced amino acid transport and protein synthesis [3, 6, 7], they can be limited by nonspecific accumulation [3]. As such, novel PET radiotracers with improved performance characteristics for functional imaging of glioma are urgently needed.

Representing a different functional target is the translocator protein (TSPO), an outer mitochondrial membrane protein that participates in numerous, native cellular processes and is highly expressed in several types of malignant cells [8, 9]. Moreover, TSPO expression can correlate with glioma tumor grade in certain settings [10] and has been linked with disease progression and diminished survival [8]. The earliest published evidence in support of the hypothesis that ligands targeting TSPO could be used to detect and grade human brain tumors emerged more than twenty years ago [11–13], easily predating molecular studies seeking to elucidate potential roles of TSPO function in tumorigenesis [9, 14, 15]. These foundational studies demonstrated the utility of [^11^C](*R*)-*N*-methyl-*N*-(1-methylpropyl)-1-(2-chlorophenyl)-isoquinoline-3-carboxamide ([^11^C]PK11195) as a radiotracer for targeted PET imaging of TSPO within glioma [16–18]. [^11^C]PK11195 exhibited relatively modest increased uptake in tumor tissue compared to normal brain, despite large differences in relative TSPO expression and high levels of non-displaceable ligand binding.

Our lab focuses on development of novel TSPO ligands with improved characteristics for glioma imaging. Towards this goal, we have evaluated [^18^F]PBR06 [19] and [^18^F]DPA-714 [20] in preclinical models of glioma, in addition to novel ligands such as [^18^F]VUIIS1008 and a fluorinated analog of SSR180575 [21–24]. These proof-of-principle imaging studies demonstrated the potential of TSPO PET to: (1) quantify TSPO levels in tumors and normal brain; (2) visualize a tumor within the normal brain.

In the current study, we demonstrate by immunohistochemical staining that TSPO is highly expressed in human glioma and correlates with grade. In preclinical studies featuring human brain tumor xenotransplants, focal uptake of [^18^F]PBR06 in the tumor core and infiltrated white matter tracts of the contralateral hemisphere was detectable by dynamic PET imaging. Notably, this infiltrative growth was not detectable by T_2_-weighted MRI, suggesting that TSPO PET has the potential to address an unmet clinical need in this setting. Furthermore, comparison to [^18^F]FDG in a preliminary study showed a level of preferential uptake of [^18^F]PBR06 within the tumor tissue that warrants further study. The preclinical studies reported herein suggest that TSPO represents a promising target in human glioma imaging for detection, staging, and therapy.

## Materials and Methods

### Human Tissue Procurement

Brain tumor specimens for the generation of a tissue microarray and xenografts were obtained from patients treated at Vanderbilt Medical Center in accordance with Vanderbilt University Institutional Review Board approval (IRB #030372). The Vanderbilt University Institutional Review Board approved the compilation of samples into a tissue microarray (IRB# 090081). All samples included in the study were derived from patients who had given written consent for tissue collection and this consent method was IRB approved. Primary brain tumors were phenotyped and graded by a neuropathologist using World Health Organization criteria.

### Tissue Microarray (TMA)

Immunohistochemistry for TSPO was performed on TMAs containing three tissue cores (1 mm diameter) from each of the 37 high-grade glioma specimens (33 grade IV and 4 grade III), as well as each of the 17 low-grade pilocytic astrocytoma specimens. Five-micron thick sections were incubated with a primary monoclonal antibody for TSPO (Novus, NB100-41398, 1:1500 dilution). Briefly, tissues were deparaffinized, rehydrated, and antigen retrieval performed using a citrate buffer solution (pH 6.0) applied for 15 minutes at 105°C, followed by a 10-minute cool-down to room temperature. A solution of 3% H_2_O_2_ was used to eliminate endogenous peroxidase activity followed by blocking with a serum-free protein blocking reagent for 20 minutes. For detection of primary antibodies, tissue sections were incubated for 60 minutes at room temperature. Subsequently, samples were incubated for 30 minutes utilizing the Envision+ System-HRP Labeled Polymer detection method. Staining was completed after incubation with a DAB substrate-chromagen solution. For each tumor core on the TMA, TSPO staining was scored on an ordinal intensity scale ranging from 0 (no expression) to 3. For each tumor, the average score for TSPO immunostaining was calculated. A patient was considered “low expressing” if the average score of the three cores was less than 1, “moderate expressing” if the average score was 1–2, and “high expressing” if the average score was greater than 2”.

### Animals and Housing

The Vanderbilt University Institutional Animal Care and Use Committee approved all studies involving the use of animals (Approval number M/09/286). Adult male athymic nude rats, weighing approximately 250 g, were obtained from Harlan Laboratories (Indianapolis, IN). Humane endpoints were defined as a loss of more that 10% of body mass, a tumor greater than 1.5 cm for a mouse or 3.0 cm for a rat, or inability to ambulate or rise for food and water.

### Orthotopic Xenotransplantation

To establish a preclinical model suitable for small-animal imaging and relevant to the highly infiltrative growth of malignant gliomas, primary xenografts were established orthotopically in NOD/SCID mice [25, 26]. Xenografted mice were euthanized and the harvested brains placed in a slicer matrix (Zivic Instruments, Pittsburgh, PA). Xenografted tumor was then dissected from a 1-mm coronal slice and dissociated with papain (Worthington Biochemical Corporation; Lakewood, NJ). Three high-grade gliomas were passaged into the rats, one WHO grade III astrocytoma (AA 17991) and two WHO grade IV glioblastomas (GBM 12055 and GBM 17182). Dissociated glioma cells (2x10^5^–3x10^5^) were transplanted into the right striatum of athymic nude rats. For transplant, rats were anesthetized with isoflurane and securely placed on a stereotactic frame. Using aseptic surgical procedures, an incision was made in the scalp and a small burr-hole drilled 2.5 mm lateral to the bregma. Glioma cells were implanted 3.5 mm into the right striatum using a Hamilton syringe. Following xenotransplantation, rats were observed for symptoms of tumor engraftment (sustained weight loss) and then subjected to small animal imaging (T_2_-weighted MRI and dynamic PET/CT) and immunohistochemical analysis.

### MR Imaging

MRI was used to localize tumors as previously published [19]. In brief, rats were secured in a prone position in a 63 mm inner diameter radiofrequency (RF) coil and placed in a Varian 4.7T horizontal bore imaging system (Varian Inc., Palo Alto, CA). A constant body temperature of 37°C was maintained using heated airflow. An initial multislice gradient echo imaging sequence [repetition time (TR) = 150 ms; echo time (TE) = 3.5 ms; 128 x 128 matrix, 40 x 40 mm^2^ FOV; 2 mm slice thickness] was used to acquire seven slices in each imaging plane (axial, coronal, sagittal) for proper positioning of subsequent scans. A multislice T_2_-weighted fast-spin echo scan with 8 echoes and 8.6 ms echo spacing was then collected with TR = 2000 ms, 32 x 32 mm^2^ FOV, 128 x 128 matrix, number of acquisitions = 16, and 8 coronal slices of 2-mm thickness. The same anatomical slices were then imaged at the same field of view (FOV) and resolution using a diffusion-weighted spin echo sequence [TR = 2000 ms; TE = 35.4 ms; number of acquisitions = 8; δ = 4 ms; Δ = 25 ms] at b-values of 0 and 600 s/mm^2^.

### Radiotracers

[^18^F]PBR06 was prepared according to published methods [19]. Briefly, using a commercial apparatus (TRACERlab FX_F-N_, GE Medical Systems, USA), aqueous [^18^F]fluoride ion (~ 111 GBq) was dried by iterative cycles of addition and evaporation of acetonitrile, followed by complexation with K^+^-K^+^-2.2.2/K_2_CO_3_. The complex was then reacted with *N*-bromoacetyl-*N*-(2,5-dimethoxybenzyl)-2-phenoxyaniline (0.8–1.2 mg) at 100°C for 20 minutes. [^18^F]PBR06 was purified using reversed-phase HPLC (C18, Dynamax 250 x 21.4 mm; Varian), eluting at 8.0 mL/min with 15 mM NaH_2_PO_4_ buffer (pH 6.7) and ethanol (47.5:52.5, v/v). [^18^F]PBR06 was collected directly into 140 mL of water (deionized), passed through a C18 Sep-Pak, and eluted with ethanol (1.0 mL), then saline (9.0 mL), into a sterile flask. Average radiochemical purity was >98%, with an average specific activity of 110 TBq/mmol (± 28 TBq/mmol). [^18^F]FDG was purchased from PETNet Solutions Inc. (Nashville, TN) and had an average reported radiochemical purity of 98.5% and an average reported activity of ≥ 37 TBq/mL.

### PET/CT Imaging

PET imaging was performed within 24 hours of MR imaging for rats with confirmed tumors. Tumor-bearing rats were administered ~ 70–100 MBq/0.2 mL [^18^F]PBR06 *via* a jugular catheter while in a microPET Focus 220 (Siemens Preclinical Solutions; Knoxville, TN). Dynamic images (90 min) were collected, followed by a computed tomography (CT) scan (microCAT II, Siemens Preclinical Solutions) for attenuation correction. For displacement studies, ‘cold’/nonradioactive PBR06 (10 mg/kg) was injected *via* jugular catheter 35 minutes after radiotracer administration. The dynamic acquisition was divided into twelve, five-second frames for the first minute, followed by 89 sixty-second frames for the duration of the scan. Data from all possible lines of response (LOR) were saved in the list mode raw data format. The raw data was then binned into 3D sinograms with a span of 3 and ring difference of 47. The images were reconstructed into transaxial slices (128 x 128 x 95) with voxel sizes of 0.095 x 0.095 x 0.08 cm^3^, after applying scatter and attenuation corrections, using an iterative ordered subsets expectation maximization (OS-EM 2D) algorithm with 16 subsets and 4 iterations. Attenuation correction was accomplished by generating an attenuation map (sinogram) from the CT image. The CT image was first co-registered with the microPET image, segmented into air, soft tissue, and bone, and then projected into sinograms with a span of 47 and ring difference of 23.

Twenty-four hours after the [^18^F]PBR06 PET scan, the same rat (GBM 17182) was imaged with [^18^F]FDG-PET. Animal handling procedures for [^18^F]FDG-PET imaging were similar to published protocols [27, 28]. In addition, prior to imaging, the animal was fasted overnight and allowed to acclimate to the PET imaging facility. [^18^F]FDG (34 MBq/0.2 mL) was administered *via* a jugular catheter, followed by a 40-minute uptake period in which the rat was conscious and free to move about. Then, a 20-minute static acquisition PET scan was collected in a microPET Focus 220. This was immediately followed with a CT scan for attenuation correction. PET images were reconstructed using the OS-EM 2D algorithm.

### Image Analysis

Time-activity curves were generated by manually segmenting three-dimensional volumes of interest over tumor and contralateral brain using ASIPro (Siemens Preclinical Solutions), avoiding areas of central necrosis if present. Volumes of interest were manually drawn on PET images to determine regions of interest (ASIPro). A rigid registration of static anatomical features was carried out by manually overlaying [^18^F]PBR06 PET images onto the MR images.

### Preclinical Immunohistochemical Analysis

Whole rat brains were harvested and fixed in 10% formalin for 48 hours, followed by paraffin embedding. Tissue sections (5.0 μm thickness) were processed for antigen retrieval and immunohistochemistry as previously described [26]. For detection of engrafted human cells, sections were stained with a human-specific vimentin antibody (MAb clone Vim 3B4, 1:200; Dako, Carpinteria, CA). Immunoenzymatic detection was achieved with the HiDef HRP Polymer System (Cell Marque, Rocklin, CA) according to the manufacturer’s instructions, using 3,3-diaminobenzidine as the chromogen.

## Results

### TSPO Expression in Human Glioma Specimens

**Fig 1:**
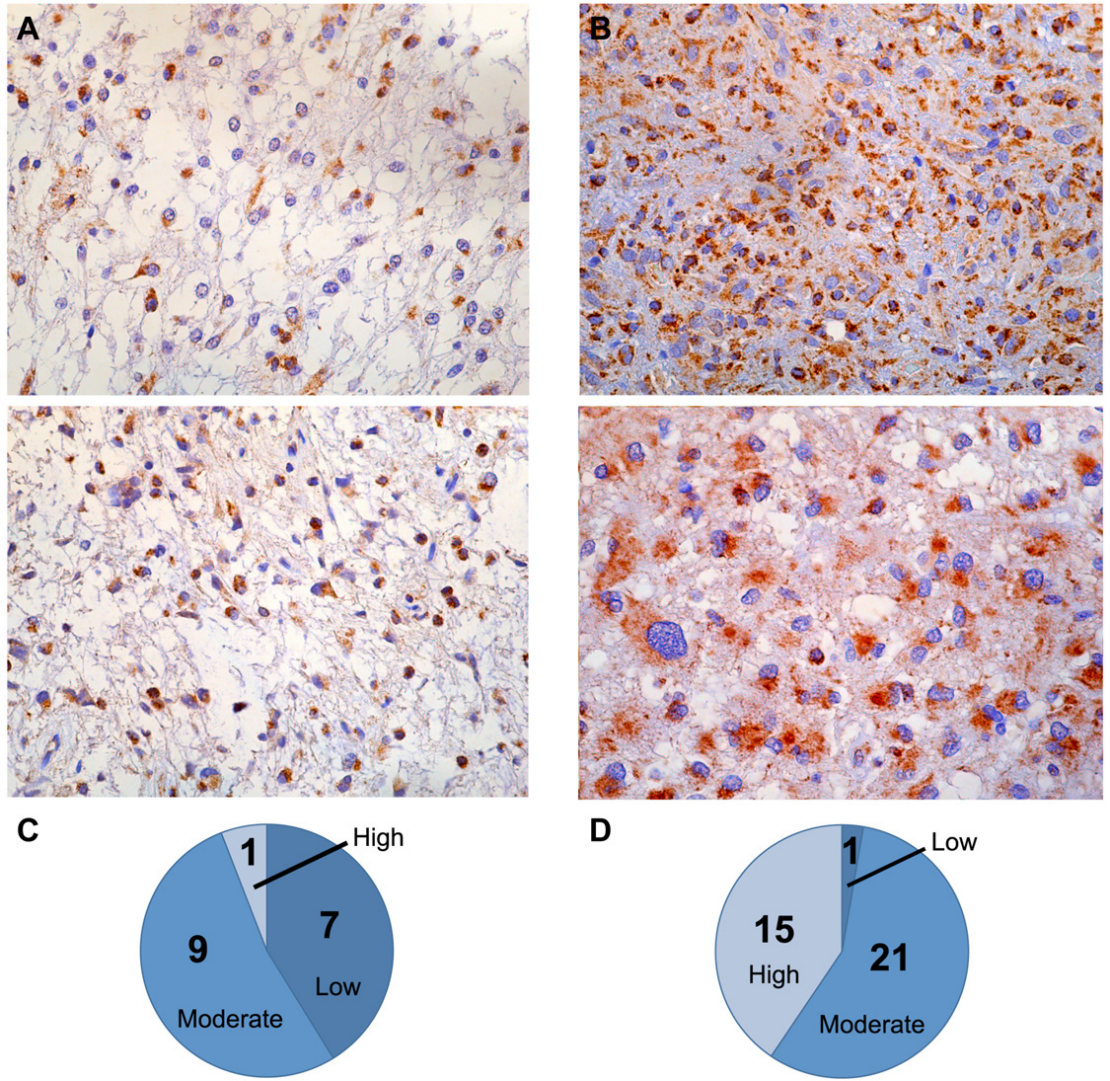
Human tumor microarrays (TMAs). Pilocytic astrocytoma TSPO immunohistochemistry (A) and TSPO immunohistochemical scoring (C). Glioblastoma multiforme TSPO immunohistochemistry (B) and TSPO immunohistochemical scoring (D).

TSPO immunoreactivity was evaluated in tumor microarrays (TMAs) of pilocytic astrocytoma (WHO grade I) and high-grade (WHO grades III and IV) glioma (Fig 1). In tumor cells, TSPO appeared to be confined to the cytoplasm and occasionally the nucleus. For the TMA generated from patients with pilocytic astrocytoma, immunohistochemistry (IHC) indicated a low level of TSPO expression, with 16/17 of the specimens scoring as low to moderate, and only 1/17 of the specimens exhibiting high TSPO levels (Fig 1A). In contrast, higher levels of TSPO expression were detected by IHC in the TMA containing high-grade, malignant glioma specimens. TSPO expression levels were scored as high in 15/37 of the samples, with 21/37 scored as moderate, and 1/37 as low (Fig 1B).

### A Preclinical Imaging Model for Infiltrative Glioma Growth

**Fig 2:**
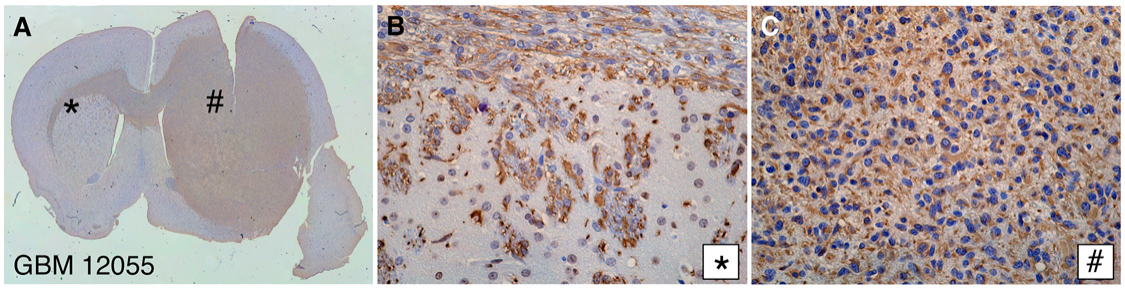
Preclinical model of glioma: Human xenotransplantation into athymic rats. Primary human grade IV glioblastoma xenotransplant, vimentin immunohistochemistry: (A) Gross; (B) Pencil fibers (40X); (C) Tumor (40X).

We previously demonstrated that high-affinity TSPO radiotracers may be used to visualize gliomas by PET imaging in rats bearing C6 glioma cell allografts [19–23]. As with other animal models utilizing glioma cell lines cultured in serum (secondary xenografts), infiltrative growth is not maintained in C6 glioma allografts. In contrast, the infiltrative phenotype is recapitulated in primary xenografts in which patient glioma cells are not cultured *in vitro* prior to transplantation [29]. We developed a preclinical rat model in which primary human gliomas were initially established orthotopically in NOD/SCID mice, and then passaged into the right striatum of athymic rats. For each of the three primary xenograft lines, IHC with a human-specific vimentin antibody demonstrated dense tumor growth within the injected striatum and infiltrative growth along myelinated fiber tracts (the corpus callosum and the striatopallidal fibers or “pencil fibers”) in the contralateral hemisphere (Fig 2). Fig 2A–2C show the gross and detailed vimentin IHC of the grade IV glioblastoma (GBM 12055) model.

### PET Imaging of Glioma and Infiltrative Glioma Growth with [^18^F]PBR06

**Fig 3:**
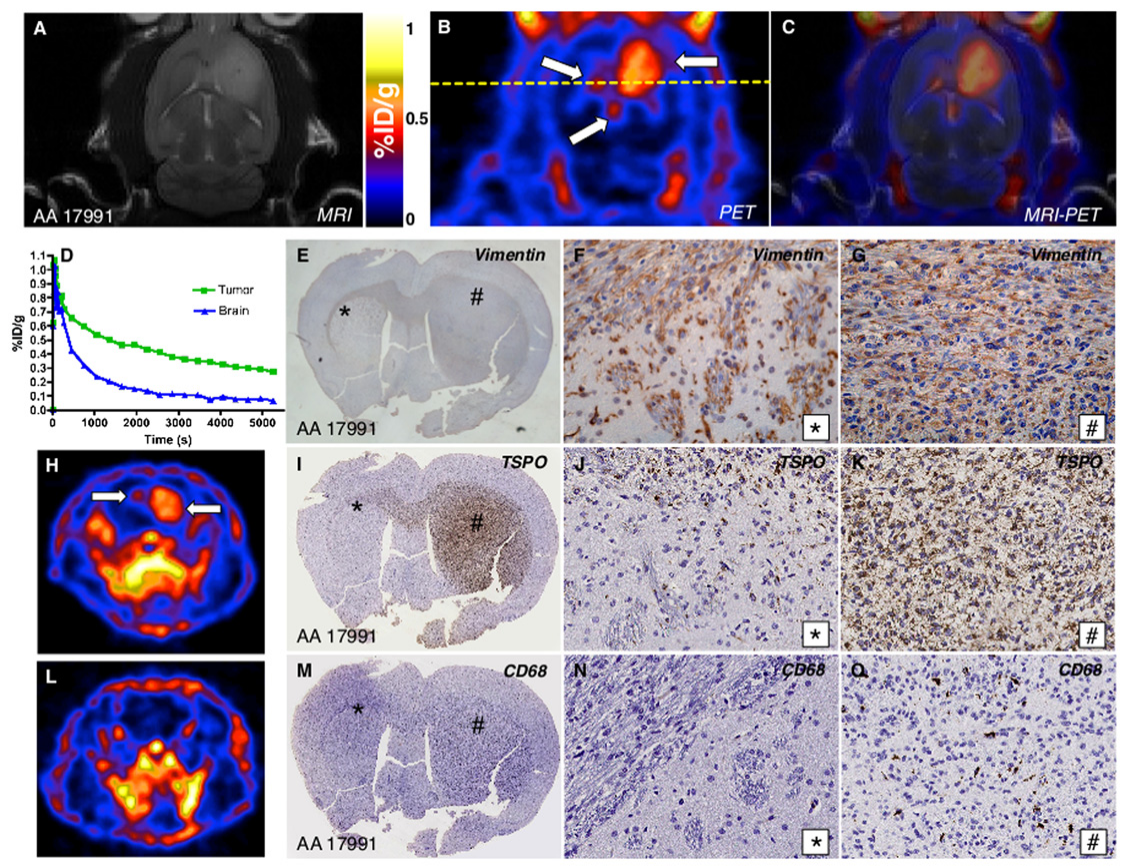
Primary human grade III astrocytoma xenotransplant. (A) T_2_-weighted MRI (coronal) visualizes advanced tumor in the right hemisphere of the brain. (B) Correlative dynamic PET image (coronal) of same advanced tumor in the right hemisphere of the brain, with [^18^F]PBR06 uptake primarily confined to the tumor and co-localizing with tumor tissue visualized by MRI. Top arrows indicate tumor and infiltration into left hemisphere. (C) Fused MRI (A) PET (B) image. (**D**) Time-activity curves of injected [^18^F]PBR06 in tumor (*green*) and contralateral brain (*blue*). Correlative vimentin immunohistochemistry: (E) Gross; (F) Tumor + White Matter Tract (40X); (G) Tumor (40X). (H) Correlative dynamic PET image, axial view along yellow line in (B); arrows indicate tumor and infiltration into left hemisphere. Correlative TSPO immunohistochemistry: (I) Gross; (J) Tumor + White Matter Tract (40X); (K) Tumor (40X). (L) Dynamic PET image (axial) of control cohort. Correlative CD68 immunohistochemistry: (M) Gross; (N) Tumor + White Matter Tract (40X); (O) Tumor (40X).

**Fig 4:**
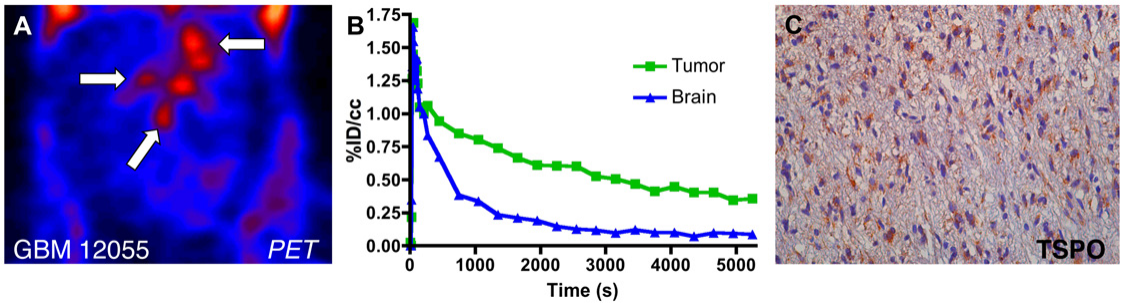
Primary human grade IV glioblastoma xenotransplant. (A) Dynamic PET image (*coronal*) of advanced tumor in the right hemisphere of the brain with [^18^F]PBR06 uptake confined primarily to the tumor. Arrows indicate tumor and infiltration into left hemisphere. (B) Time-activity curves of injected [^18^F]PBR06 in tumor (*green*) and contralateral brain (*blue*) [30]. (C) Correlative TSPO immunohistochemistry, Tumor + White Matter Tract (40X).

Prior to dynamic PET imaging of AA 17991 (Grade III astrocytoma) engrafted rats, tumors were localized with T_2_-weighted MRI. Hyperintense T_2_-weighted signal indicative of advanced tumor growth were observed in the right hemisphere where the tumors cells were implanted (Fig 3A). Subsequent PET imaging revealed intense [^18^F]PBR06 uptake not only within the tumor implanted in the striatum of the right hemisphere (Fig 3B, right arrow), but also distally in the left corpus callosum (Fig 3B, left arrow), as revealed by the MRI-PET overlay (Fig 3C). The signal indicated by the bottom arrow in Fig 3B is likely due to [^18^F]PBR06 binding of the dorsal third ventricle, an area with known high TSPO expression and high [^3^H]PK11195 binding [30]. Time-activity curves (TACs) of the [^18^F]PBR06 signal in the tumor core of the right hemisphere (*green*) and in uninvolved regions of posterior contralateral hemisphere (*blue*) [31] suggest preferential uptake and retention of the probe within the tumor (Fig 3D). Vimentin IHC (Fig 3E) demonstrated analogous features to the GBM 12055 model (Fig 2), with dense tumor growth within the injected striatum (Fig 3G) and infiltrative growth along myelinated fiber tracts in the contralateral hemisphere (Fig 3F). A view along the axial plane (yellow line in Fig 3B) also showed strong [^18^F]PBR06 uptake in the tumor (right arrow) and lower uptake in the left corpus callosum (left arrow) (Fig 3H). Confirming that this signal represents selective uptake of probe by malignant cells, correlative TSPO IHC was carried out. Fig 3I (gross view) shows strong expression of TSPO at the site of tumor injection in the right striatum (Fig 3K), which decreases in intensity as it progresses between the two hemispheres and into the myelinated fiber tracts in the left hemisphere (Fig 3J). Of note when comparing the TSPO histology to Fig 3H is the apparent lack of corresponding [^18^F]PBR06 signal between the two hemispheres. This is a direct result of the resolution difference between PET (mm) and microscopy (μm) [32]. Comparison of the [^18^F]PBR06 signal in the tumor model (Fig 3H) to a control (Fig 3L) reveals minimal uptake of the probe under healthy conditions. Confirming that the [^18^F]PBR06 signal (Fig 3H) represented preferential uptake of the probe by neoplastic cells over glioma-associated microglia/macrophages (GAMs), correlative CD68 IHC (Fig 3M) demonstrated nominal GAM expression within the myelinated fiber tracts of the contralateral hemisphere (Fig 3N) and in the tumor core (Fig 3O). This selective uptake of [^18^F]PBR06 by TSPO in neoplastic cells in the engrafted tumor core, as well as infiltrative components, was further corroborated in the GBM 12055 model (Fig 4A–4C).

### *In vivo* Displacement of [^18^F]PBR06

**Fig 5:**
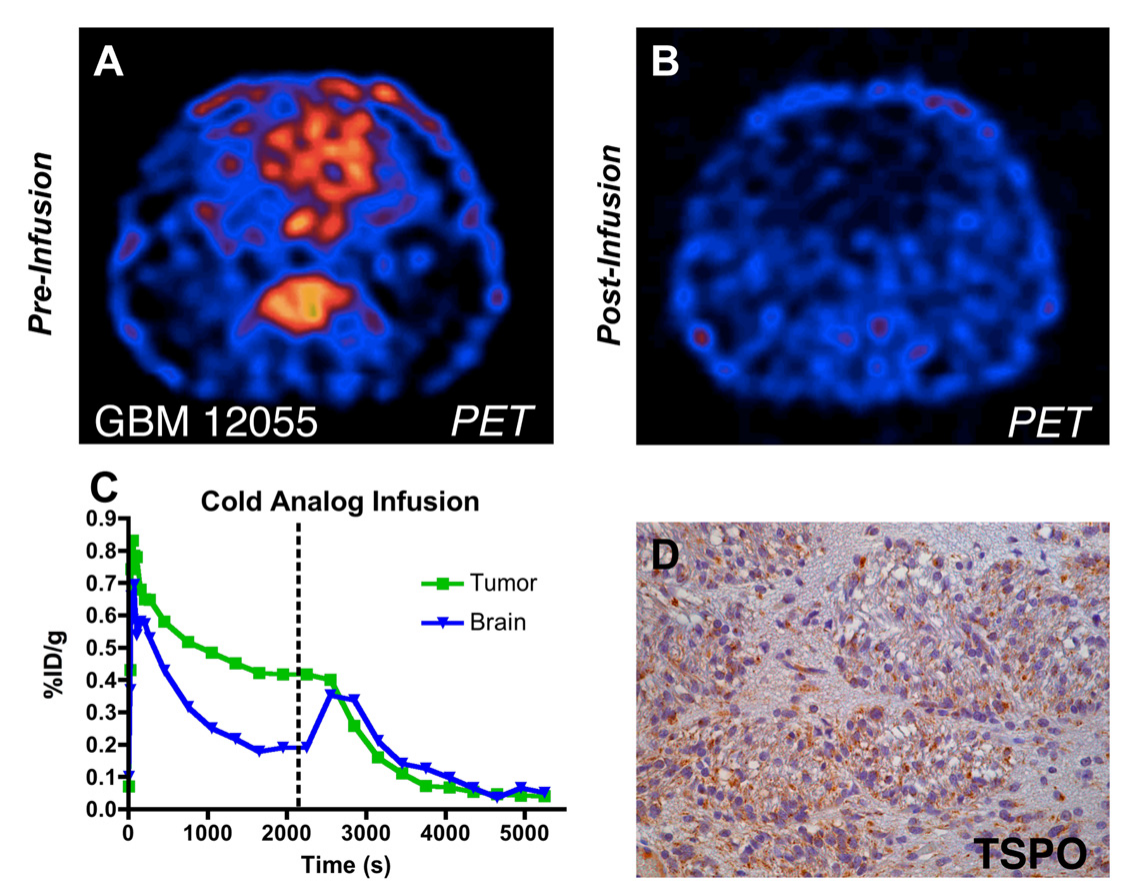
[^18^F]PBR06 selectivity in primary human grade IV glioblastoma xenotransplant. (A) Dynamic PET image (axial) pre-infusion of cold analog, with [^18^F]PBR06 uptake primarily confined to the tumor. (B) Dynamic PET image (axial) post-infusion, showing nearly total displacement of [^18^F]PBR06. (C) Time-activity curves of injected [^18^F]PBR06 in tumor (*green*) and contralateral brain (*blue*). (D) Correlative TSPO immunohistochemistry, Tumor + White Matter Tract (40X).

To assess the specificity of [^18^F]PBR06 binding *in vivo*, displacement studies using non-radioactive, ‘cold’ PBR06 were carried out in rats bearing a tumor from the GBM 12055 cohort. Excess PBR06 (10 mg/kg) was administered intravenously 35 minutes after [^18^F]PBR06 injection during the dynamic PET study. Summation of the first 35 minutes of the PET scan, prior to injection of cold PBR06 (0–35 min), demonstrated typical uptake characteristics of [^18^F]PBR06 (Fig 5A). Displacement of [^18^F]PBR06 with cold PBR06 was measured by summation of the final 30 minutes of the PET scan (60–90 min) (Fig 5B). Time–activity curve analysis showed that tumor activity was reduced to less than 10% of its peak uptake following injection of cold PBR06, indicating a displaceable binding level of approximately 90% (Fig 5C). Confirming that the [^18^F]PBR06 signal represented selective uptake of probe by malignant cells, correlative IHC (Fig 5D) demonstrated strong expression of TSPO within the myelinated fiber tracts and tumor. In further support of [^18^F]PBR06 binding specificity, a corresponding transient influx of displaced radiotracer from other TSPO-rich organs, such as kidney, heart, and gonads into normal brain was observed during the cold PBR06 infusion phase (60–90 min), which subsequently washed out rapidly [23]. After displacement, residual brain activity reached levels similar to the normal, age-matched control (S1 Fig).

### Comparison of [^18^F]PBR06 and [^18^F]FDG PET Imaging

**Fig 6:**
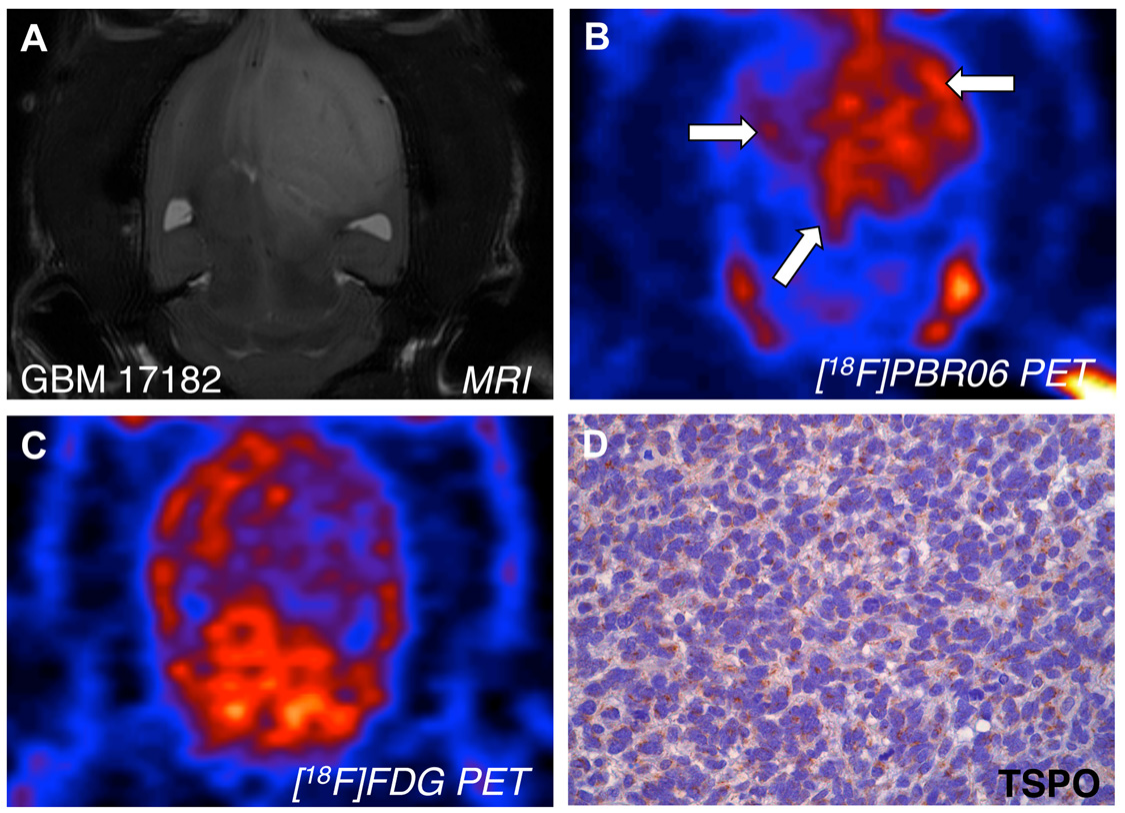
Side-by-side PET comparison of [^18^F]PBR06 and [^18^F]FDG in a primary human grade III astrocytoma xenotransplant. (A) T_2_-weighted MRI (*coronal*) visualizes advanced tumor in the right hemisphere of the brain. (B) [^18^F]PBR06 uptake (*coronal*) is primarily confined to the tumor and co-localizes with tumor tissue visualized by MRI. Arrows indicate tumor and infiltration into left hemisphere. (C) [^18^F]FDG uptake (*coronal*) in normal brain is higher than tumor tissue, resulting in poor imaging contrast. (D) Correlative TSPO immunohistochemistry, Tumor + White Matter Tract (40X).

To determine the potential utility of TSPO imaging in relation to another PET tracer currently used for malignant glioma, [^18^F]PBR06 was compared to [^18^F]FDG in a human grade III astrocytoma (GBM 17182) xenotransplant (Fig 6). Following T_2_-weighted MRI localization of the tumor (Fig 6A), PET imaging with [^18^F]PBR06 (Fig 6B) revealed uptake of the probe within the region of T_2_ signal changes (right arrow) and infiltrative growth in the contralateral hemisphere (left arrowheads). In contrast, [^18^F]FDG PET imaging demonstrated higher uptake in the normal brain relative the region of T_2_ signal abnormality, resulting in poor image contrast (Fig 6C). IHC confirmed robust TSPO expression in the tumor (Fig 6D).

## Discussion

Neuroimaging serves many critical roles in the management of patients with malignant glioma by guiding diagnostic procedures, planning radiation therapy, and assessing treatment outcomes. Clinically, MRI remains the most useful imaging modality despite its lack of sensitivity and specificity for detecting infiltrative and residual disease, as well as radiation necrosis. Of note, magnetic resonance spectroscopy (MRS), an adjunct of MRI, has the potential to reflect metabolism in human glioma through quantification of biomarkers of tumor metabolism, membrane turnover, and proliferation as ratios between glucose, choline, and creatine, respectively. However, a recent meta-analysis showed that MRS alone, using such metabolite ratios, only demonstrates moderate diagnostic performance in differentiating glioma recurrence from radiation necrosis. As such, it is recommended this technique be combined with other imaging technologies to improve diagnostic accuracy, with future research efforts directed towards multimodal imaging trials and multicenter trials [33].

Accordingly, molecular imaging with PET stands as a viable alternative, though currently only utilized to a limited extent. Beyond [^18^F]FDG, the only reimbursed tracer for oncology, other tracers such as [^11^C]MET, [^18^F]FET, and [^18^F]DOPA have been explored to various extents with mixed results and limitations related to tracer half-life, background signal, and/or blood brain barrier permeability [3].

The studies presented herein sought to extend our original observation that [^18^F]PBR06 may possess utility in targeting TSPO for brain tumor imaging [19]. Focusing primarily on comparing low grade to high grade, we constructed a human glioma TMA consisting of grade I and grade III/IV samples. While TSPO staining was detected in all tumor specimens, the high grade samples overall showed a much greater presence of TSPO than that of the low. The xenotransplant models developed for this study using high-grade (III, IV) clinical specimens, in addition to being more clinically relevant than less infiltrative C6 glioma models, allowed evaluation of imaging metrics within the context of microscopic tumor infiltration. As with the original mouse model [25], important pathological features of malignant glioma were recapitulated in the rat model, including infiltrative growth and invasion of the corpus callosum to involve the contralateral hemisphere. Upon orthotopic xenotransplantation, the tumors retained not only the histopathological features of the original specimen, but also key molecular features [26], including elevated TSPO expression. This data agrees with previous work of Miettinen *et al*., who in addition to reporting the close association of TSPO expression with cell proliferation and tumor malignancy, also noted that tumors expressing high levels of TSPO showed a tendency for poor survival [34].

Molecular imaging studies with the TSPO PET ligand [^18^F]PBR06 revealed selective and targeted uptake not only in engrafted tumor, but infiltrative growth in the opposite hemisphere previously undetected by our T_2_-weighted MRI scans. High specificity of [^18^F]PBR06 for TSPO within the target tissue was confirmed when 90% of the radiotracer was displaced by unlabeled, ‘cold’ PBR06. Correlative IHC confirmed robust expression of TSPO in both tumor and the infiltrated fiber tracts of the contralateral hemisphere. Moreover, correlative CD68 IHC demonstrated nominal glioma-associated microglia/macrophage (GAM) expression within the same areas. Together, these verified that the [^18^F]PBR06 signal represented preferential uptake of the probe by TSPO in neoplastic cells over GAMs. These results agree with Su *et al*.’s recent work that looked at TSPO expression in tumor tissue and GAMs, wherein they demonstrated using [^11^C]PK11195 PET that TSPO is predominantly expressed in neoplastic cells, with GAMs only partially contributing to the PET signal [35]. Exploratory studies comparing [^18^F]PBR06 to the most widely applied radiotracer for brain tumor assessment, [^18^F]FDG, showed a level of preferential uptake of [^18^F]PBR06 within the tumor tissue that merits further investigation.

## Conclusion

The objective of this preliminary study was to evaluate the correlation between TSPO expression and glioma grade (low, high) in clinical samples, and to explore the utility of [^18^F]PBR06 to assess TSPO expression in a clinically relevant rat glioma model. In the clinical samples, TSPO levels proved highest in grade III and IV infiltrating gliomas, whereas low-grade tumors (grade I pilocytic astrocytoma) expressed TSPO levels just above non-tumor background, suggesting that TSPO PET could potentially serve as a non-invasive indicator of glioma grade and better evaluate disease extent, especially white-matter infiltration, compared to current standard-of-care imaging. Preliminary PET imaging with the developed rat model using [^18^F]PBR06 revealed infiltrative glioma growth previously undetected by traditional MRI. Continued study and development of both probe and model appear warranted. Using this model, future high-affinity TSPO ligands could be vetted for translation to human cancer imaging studies and ultimately the clinic, where surgical resection could be better guided by more accurate enhancement of invasive tumor margins. Determining whether TSPO PET better delineates the true extent of tumor involvement as compared to MRI in patients with malignant glioma warrants further study, as it would have important clinical implications for detection, therapy planning, and post-treatment monitoring.

## Supporting Information

S1 Fig Normal, age-matched control. (A) Dynamic [^18^F]PBR06 PET image (coronal). (B) Time-activity curve of injected [^18^F]PBR06 in brain.
(TIF)

## References

[1] Chen W, Cloughesy T, Kamdar N, Satyamurthy N, Bergsneider M, Liau L, et al Imaging proliferation in brain tumors with F-18-FLT PET: Comparison with F-18-FDG. Journal of Nuclear Medicine. 2005;46(6):945–52. . 15937304

[2] Nihashi T, Dahabreh IJ, Terasawa T. PET in the clinical management of glioma: evidence map. AJR American journal of roentgenology. 2013;200(6):W654–60. Epub 2013/05/25. 10.2214/AJR.12.9168 . 23701099

[3] la Fougere C, Suchorska B, Bartenstein P, Kreth FW, Tonn JC. Molecular imaging of gliomas with PET: opportunities and limitations. Neuro-oncology. 2011;13(8):806–19. Epub 2011/07/16. 10.1093/neuonc/nor054 21757446 PMC3145468

[4] Santra A, Kumar R, Sharma P, Bal C, Julka PK, Malhotra A. F-18 FDG PET-CT for predicting survival in patients with recurrent glioma: a prospective study. Neuroradiology. 2011 Epub 2011/07/09. 10.1007/s00234-011-0898-3 . 21739222

[5] Dhermain FG, Hau P, Lanfermann H, Jacobs AH, van den Bent MJ. Advanced MRI and PET imaging for assessment of treatment response in patients with gliomas. The Lancet Neurology. 2010;9(9):906–20. Epub 2010/08/14. 10.1016/S1474-4422(10)70181-2 . 20705518

[6] Goldman S, Pirotte BJ. Brain tumors. Methods Mol Biol. 2011;727:291–315. Epub 2011/02/19. 10.1007/978-1-61779-062-1_16 . 21331940

[7] Pirotte B, Goldman S, Massager N, David P, Wikler D, Vandesteene A, et al Comparison of 18F-FDG and 11C-methionine for PET-guided stereotactic brain biopsy of gliomas. Journal of nuclear medicine: official publication, Society of Nuclear Medicine. 2004;45(8):1293–8. Epub 2004/08/10. doi: 45/8/1293 [pii]. . 15299051

[8] Batarseh A, Papadopoulos V. Regulation of translocator protein 18 kDa (TSPO) expression in health and disease states. Molecular and cellular endocrinology. 2010;327(1–2):1–12. Epub 2010/07/06. 10.1016/j.mce.2010.06.013 20600583 PMC2922062

[9] Papadopoulos V, Baraldi M, Guilarte TR, Knudsen TB, Lacapere JJ, Lindemann P, et al Translocator protein (18 kDa): new nomenclature for the peripheral-type benzodiazepine receptor based on its structure and molecular function. Trends Pharmacol Sci. 2006;27(8):402–9. Epub 2006/07/11. doi: S0165-6147(06)00153-2 [pii] 10.1016/j.tips.2006.06.005 . 16822554

[10] Vlodavsky E, Soustiel JF. Immunohistochemical expression of peripheral benzodiazepine receptors in human astrocytomas and its correlation with grade of malignancy, proliferation, apoptosis and survival. J Neurooncol. 2007;81(1):1–7. Epub 2006/07/27. 10.1007/s11060-006-9199-9 . 16868661

[11] Starostarubinstein S, Ciliax BJ, Penney JB, Mckeever P, Young AB. Imaging of a Glioma Using Peripheral Benzodiazepine Receptor Ligands. Proceedings of the National Academy of Sciences of the United States of America. 1987;84(3):891–5. 10.1073/Pnas.84.3.891 . 3027710 PMC304322

[12] Olson JM, Junck L, Young AB, Penney JB, Mancini WR. Isoquinoline and peripheral-type benzodiazepine binding in gliomas: implications for diagnostic imaging. Cancer research. 1988;48(20):5837–41. Epub 1988/10/15. . 3262414

[13] Black KL, Ikezaki K, Toga AW. Imaging of brain tumors using peripheral benzodiazepine receptor ligands. Journal of neurosurgery. 1989;71(1):113–8. Epub 1989/07/01. 10.3171/jns.1989.71.1.0113 . 2544689

[14] Hardwick M, Fertikh D, Culty M, Li H, Vidic B, Papadopoulos V. Peripheral-type benzodiazepine receptor (PBR) in human breast cancer: correlation of breast cancer cell aggressive phenotype with PBR expression, nuclear localization, and PBR-mediated cell proliferation and nuclear transport of cholesterol. Cancer research. 1999;59(4):831–42. Epub 1999/02/24. . 10029072

[15] Batarseh A, Li J, Papadopoulos V. Protein kinase Cepsilon regulation of translocator protein (18 kDa) Tspo gene expression is mediated through a MAPK pathway targeting STAT3 and c-Jun transcription factors. Biochemistry. 2010;49(23):4766–78. Epub 2010/05/18. 10.1021/bi100020e 20469933 PMC2902160

[16] Cornu P, Benavides J, Scatton B, Hauw JJ, Philippon J. Increase in Omega-3 (Peripheral-Type Benzodiazepine) Binding-Site Densities in Different Types of Human Brain-Tumors—a Quantitative Autoradiography Study. Acta Neurochirurgica. 1992;119(1–4):146–52. . 1336303 10.1007/BF01541799

[17] Junck L, Olson JMM, Ciliax BJ, Koeppe RA, Watkins GL, Jewett DM, et al Pet Imaging of Human Gliomas with Ligands for the Peripheral Benzodiazepine Binding-Site. Annals of Neurology. 1989;26(6):752–8. . 2557794 10.1002/ana.410260611

[18] Pappata S, Cornu P, Samson Y, Prenant C, Benavides J, Scatton B, et al Pet Study of Carbon-11-Pk 11195 Binding to Peripheral Type Benzodiazepine Sites in Glioblastoma—a Case-Report. Journal of Nuclear Medicine. 1991;32(8):1608–10. . 1651383

[19] Buck JR, McKinley ET, Hight MR, Fu A, Tang D, Smith RA, et al Quantitative, preclinical PET of translocator protein expression in glioma using 18F-N-fluoroacetyl-N-(2,5-dimethoxybenzyl)-2-phenoxyaniline. Journal of nuclear medicine: official publication, Society of Nuclear Medicine. 2011;52(1):107–14. Epub 2010/12/15. doi: jnumed.110.081703 [pii] 10.2967/jnumed.110.081703 21149488 PMC3027353

[20] Tang D, Hight MR, McKinley ET, Fu A, Buck JR, Smith RA, et al Quantitative preclinical imaging of TSPO expression in glioma using N,N-diethyl-2-(2-(4-(2-18F-fluoroethoxy)phenyl)-5,7-dimethylpyrazolo[1,5-a]pyrimi din-3-yl)acetamide. Journal of nuclear medicine: official publication, Society of Nuclear Medicine. 2012;53(2):287–94. Epub 2012/01/19. 10.2967/jnumed.111.095653 22251555 PMC3391587

[21] Tang D, McKinley ET, Hight MR, Uddin MI, Harp JM, Fu A, et al Synthesis and structure-activity relationships of 5,6,7-substituted pyrazolopyrimidines: discovery of a novel TSPO PET ligand for cancer imaging. Journal of medicinal chemistry. 2013;56(8):3429–33. Epub 2013/03/26. 10.1021/jm4001874 23521048 PMC3648642

[22] Cheung YY, Nickels ML, Tang D, Buck JR, Manning HC. Facile synthesis of SSR180575 and discovery of 7-chloro-N,N,5-trimethyl-4-oxo-3(6-[(18)F]fluoropyridin-2-yl)-3,5-dihydro-4H-pyri dazino[4,5-b]indole-1-acetamide, a potent pyridazinoindole ligand for PET imaging of TSPO in cancer. Bioorg Med Chem Lett. 2014;24(18):4466–71. Epub 2014/08/31. 10.1016/j.bmcl.2014.07.091 25172419 PMC4163096

[23] Tang D, Nickels ML, Tantawy MN, Buck JR, Manning HC. Preclinical imaging evaluation of novel TSPO-PET ligand 2-(5,7-Diethyl-2-(4-(2-[(18)F]fluoroethoxy)phenyl)pyrazolo[1,5-a]pyrimidin-3-yl)- N,N-diethylacetamide ([(18)F]VUIIS1008) in glioma. Mol Imaging Biol. 2014;16(6):813–20. Epub 2014/05/23. 10.1007/s11307-014-0743-2 24845529 PMC4372299

[24] Powell AE, Vlacich G, Zhao ZY, McKinley ET, Washington MK, Manning HC, et al Inducible loss of one Apc allele in Lrig1-expressing progenitor cells results in multiple distal colonic tumors with features of familial adenomatous polyposis. American journal of physiology Gastrointestinal and liver physiology. 2014;307(1):G16–23. Epub 2014/05/17. 10.1152/ajpgi.00358.2013 24833705 PMC4080164

[25] Sarangi A, Valadez JG, Rush S, Abel TW, Thompson RC, Cooper MK. Targeted inhibition of the Hedgehog pathway in established malignant glioma xenografts enhances survival. Oncogene. 2009;28(39):3468–76. Epub 2009/07/21. doi: onc2009208 [pii] 10.1038/onc.2009.208 19617900 PMC2756306

[26] Gerardo Valadez J, Grover VK, Carter MD, Calcutt MW, Abiria SA, Lundberg CJ, et al Identification of Hedgehog pathway responsive glioblastomas by isocitrate dehydrogenase mutation. Cancer letters. 2013;328(2):297–306. Epub 2012/10/16. 10.1016/j.canlet.2012.10.002 23063752 PMC4308293

[27] Fueger BJ, Czernin J, Hildebrandt I, Tran C, Halpern BS, Stout D, et al Impact of animal handling on the results of 18F-FDG PET studies in mice. Journal of nuclear medicine: official publication, Society of Nuclear Medicine. 2006;47(6):999–1006. Epub 2006/06/03. doi: 47/6/999 [pii]. . 16741310

[28] Dandekar M, Tseng JR, Gambhir SS. Reproducibility of 18F-FDG microPET studies in mouse tumor xenografts. Journal of nuclear medicine: official publication, Society of Nuclear Medicine. 2007;48(4):602–7. Epub 2007/04/03. doi: 48/4/602 [pii]. . 17401098 10.2967/jnumed.106.036608 PMC4161128

[29] Shu Q, Wong KK, Su JM, Adesina AM, Yu LT, Tsang YT, et al Direct orthotopic transplantation of fresh surgical specimen preserves CD133+ tumor cells in clinically relevant mouse models of medulloblastoma and glioma. Stem Cells. 2008;26(6):1414–24. Epub 2008/04/12. doi: 2007–1009 [pii] 10.1634/stemcells.2007-1009 . 18403755

[30] Chen MK, Guilarte TR. Translocator protein 18 kDa (TSPO): molecular sensor of brain injury and repair. Pharmacol Ther. 2008;118(1):1–17. Epub 2008/04/01. doi: S0163-7258(08)00016-8 [pii] 10.1016/j.pharmthera.2007.12.004 18374421 PMC2453598

[31] Ouwerkerk R, Jacobs MA, Macura KJ, Wolff AC, Stearns V, Mezban SD, et al Elevated tissue sodium concentration in malignant breast lesions detected with non-invasive 23Na MRI. Breast cancer research and treatment. 2007;106(2):151–60. Epub 2007/01/30. 10.1007/s10549-006-9485-4 . 17260093

[32] Weissleder R, Pittet MJ. Imaging in the era of molecular oncology. Nature. 2008;452(7187):580–9. Epub 2008/04/04. 10.1038/nature06917 18385732 PMC2708079

[33] Zhang H, Ma L, Wang Q, Zheng X, Wu C, Xu BN. Role of magnetic resonance spectroscopy for the differentiation of recurrent glioma from radiation necrosis: a systematic review and meta-analysis. European journal of radiology. 2014;83(12):2181–9. Epub 2014/12/03. 10.1016/j.ejrad.2014.09.018 . 25452098

[34] Miettinen H, Kononen J, Haapasalo H, Helen P, Sallinen P, Harjuntausta T, et al Expression of peripheral-type benzodiazepine receptor and diazepam binding inhibitor in human astrocytomas: relationship to cell proliferation. Cancer research. 1995;55(12):2691–5. Epub 1995/06/15. . 7780986

[35] Su Z, Roncaroli F, Durrenberger PF, Coope DJ, Karabatsou K, Hinz R, et al The 18-kDa mitochondrial translocator protein in human gliomas: an 11C-(R)PK11195 PET imaging and neuropathology study. Journal of nuclear medicine: official publication, Society of Nuclear Medicine. 2015;56(4):512–7. Epub 2015/02/28. 10.2967/jnumed.114.151621 . 25722450

