# Supplementary figures and images for: Preclinical TSPO Ligand PET to Visualize Human Glioma Xenotransplants: A Preliminary Study
Source: PLoS One. 2015 Oct 30;10(10):e0141659. doi: 10.1371/journal.pone.0141659 (PMC4627825; doi:10.1371/journal.pone.0141659)

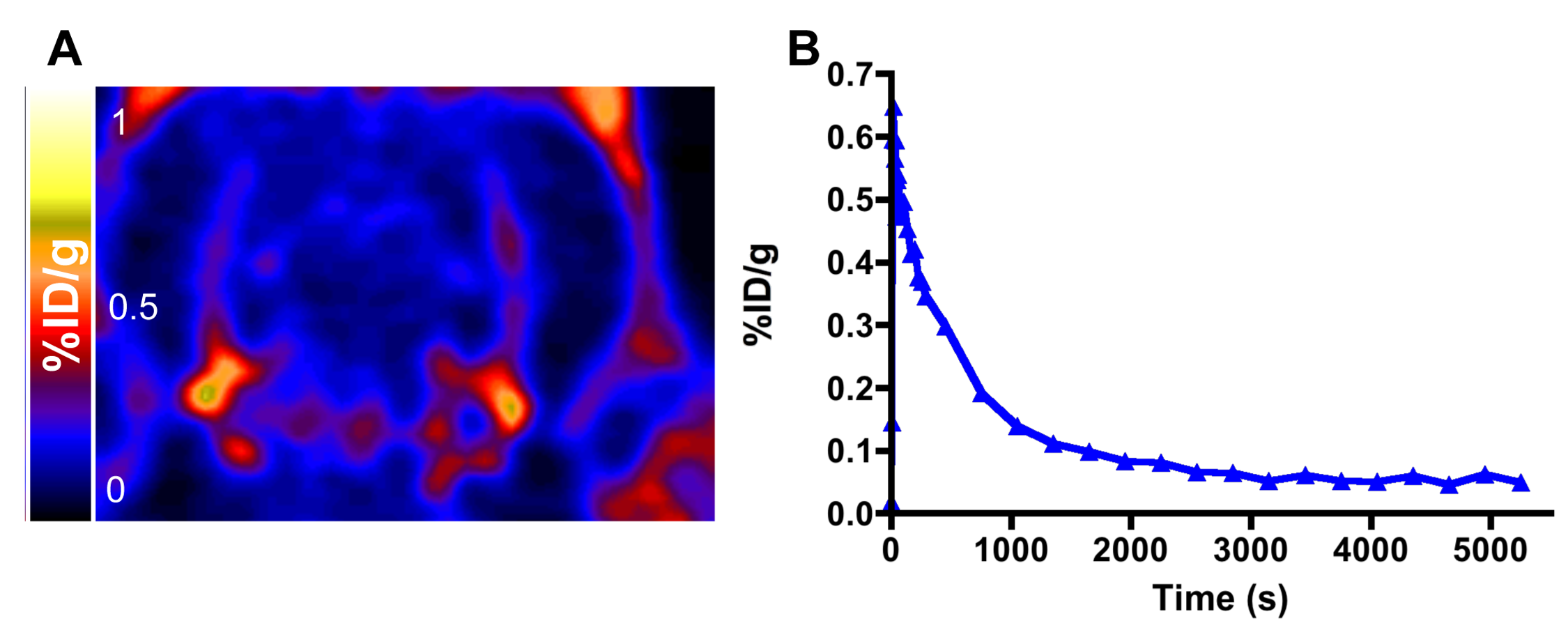

Supplement: S1 Fig — (A) Dynamic [18F]PBR06 PET image (coronal). (B) Time-activity curve of injected [18F]PBR06 in brain. (TIF) [file pone.0141659.s001.tif]
